# Socio-economic and demographic factors influencing nutritional status among early childbearing young mothers in Bangladesh

**DOI:** 10.1186/s12905-016-0338-y

**Published:** 2016-08-26

**Authors:** Ashraful Islam, Nurul Islam, Premananda Bharati, Saw Aik, Golam Hossain

**Affiliations:** 1 Research Management Centre, Faculty of Medicine, University of Malaya, Kuala Lumpur, 50603 Malaysia; 2 Department of Statistics, University of Rajshahi, Rajshahi, 6205 Bangladesh; 3 Biological Anthropology Unit, Indian Statistical Institute, 203 SH1 State highways 1, Kolkata, 700108 India; 4 Department of Orthopaedic Surgery, Faculty of Medicine, University of Malaya, Kuala Lumpur, 50603 Malaysia

**Keywords:** Early Childbearing, Young Mother, Underweight, BDHS-2011, Logistic Regression

## Abstract

**Background:**

Early childbearing influences women’s health. This study aims to examine the effects of socio-demographic factors on nutritional status of early childbearing mothers in Bangladesh based on Body Mass Index (BMI) as the indicator.

**Methods:**

Data was extracted from Bangladesh Demographic and Health Survey (BDHS)-2011. The survey was performed on 17,842 married women aged 15–49. We focused on early childbearing mothers (age ≤ 24, and who had delivered their first child ≤ 20). Mothers who were underweight (BMI ≤ 18.5 kg/m^2^) would be further classified into various grades of chronic energy deficiency (CED): mild (17.0 ≤ BMI < 18.5 kg/m^2^), moderate (16.0 ≤ BMI <17.0 kg/m^2^), and severe (BMI < 16.0 kg/m^2^). Multiple logistic regression model was used to examine the effect of socio-demographic factors on nutritional status.

**Results:**

Mean age of the mothers was 20.49 ± 2.37 years (ranged 15–24 years). The prevalence of underweight among early childbearing mothers was 32.1 % (urban 25 % and rural 35.1 %). Most of the underweight mothers had mild (62.2 %) CED, while the remaining had either moderate (25.9 %) or severe (11.9 %) CED. Multiple logistic regression analysis demonstrated that young mothers from rural areas, poor families, and those who were illiterate or with low level of education, working, and married to unemployed husband were at higher risk for being underweight. Young mothers who had non-caesarean delivered, delivered at home, or married at early age and had more than two children were also at higher risk for being underweight.

**Conclusions:**

The prevalence of underweight among early childbearing mothers in Bangladesh is very high (32.1 %), associated with the still common practice of teenage marriage. Education level, wealth index, occupation, place of residence, age at first marriage and parity were important predictors for their nutritional status. The government and non-government organizations should take initiatives to reduce the prevalence of underweight mothers in Bangladesh.

## Background

Women who become pregnant before age 20 are considered as early childbearing mothers. General wellbeing and nutritional requirements of these young mothers have recently received more attention especially in developing and under-developed countries [1]. It has been shown that early childbearing mothers were at higher risk of prenatal morbidities such as gestational diabetes, gestational hypertension and preterm labour compared to the general population [2]. The risk of pregnancy related mortality for mothers aged 15 to 19 was twice as high compared to those aged 20 and older [3]. The mortality risk would be 5–7 times higher for mothers who became pregnant before age 15 [4]. Some anthropometric and socio-economic factors had been associated with adverse health consequences among adolescent mothers [5]. The pelvic bone of young mothers may not have fully developed to accommodate the passage of the babies, increasing the risk of obstructed labour [6]. A recent longitudinal study on African-American community in Chicago reported that adolescent mothers were more likely to be unemployed, live in poverty and dependent on social welfare [7].

Body mass index (BMI) is calculated from a person’s weight and height. It is an important indicator of the nutritional status for a population. BMI value of less than or equal to 18.5 kg/m^2^ is considered as underweight, and this is a common finding among people suffering from chronic energy deficiency. Underweight women were associated with higher risk of adverse health outcomes like hip fractures [8, 9]. Pregnant mothers who were underweight have higher risk of perinatal mortality, and delivering low birth weight babies [10]. The mean age of first marriage for Bangladeshi women was 15.69 ± 2.97 years [11]. Based on a report by Kamal in 2012, the practice of teenage marriage and early childbearing were still common in Bangladesh despite substantial improvements in various Human Development indicators (HDI) [12].

In the urban areas of Bangladesh, researchers have reported that underweight was very common among ever-married non-pregnant women [13]. Prevalence of chronic energy deficiency (CED, with BMI <18.5 kg/m^2^) was very high among women from poor families in both rural and urban areas of this country (38.8 % rural, 29.7 % urban poor) [14]. More recently, Hossain et al. studied the association between BMI and socio-demographic factors among ever-married non-pregnant Bangladeshi women (aged 15–49 years) based on birth year cohorts from 1957 to 1992. They reported an increasing trend of BMI during the first sixteen years from 1957 to 1972, but a decreasing trend thereafter of Bangladeshi ever married women aged 15–49 years [15].

Information on the importance of adequate nutritional for childbearing mothers would not only help the community to understand their needs, but also indirectly help to promote the well being on their children who would eventually grow up and contribute as leaders or workforce for the nation. This is especially true in Asian communities because of the traditional role of mothers in the family and the community. Studies have already shown that nutritional status of married and unmarried women was influenced by various demographic and socio-economic factors such as age, education level, wealth index, age at first marriage, number of ever-born children, residence, religion, occupation, place of delivery and method of delivery [13, 15–17]. Two of these studies were focused on married Bangladeshi women in their reproductive age [13, 15], and two others were conducted on Indian women [16, 17]. In Bangladesh, special attention should be given to early childbearing mothers because this subgroup of the population was associated with high risk of adverse health outcome compared to mothers of older age [1]. Moreover, practice of teenage marriage remained common in Bangladesh. There has not been any published study on the general health for this group of population in Bangladesh. We therefore decided to use the nutritional status as proxy for general health, and study risk factors related to the general health status of early childbearing mothers in this country.

## Methods

Information for this cross sectional study was extracted from a sample of 17,842 married Bangladeshi women (age 15–49 years) from the Bangladesh Demographic and Health Survey (2011- BDHS). From this initial data set, we identified married Bangladeshi women age 24 and below, and selected those who had been pregnant and delivered before age 20. We considered age 24 as the cut off point for inclusion because the practice of early childbearing might still influence the health status of these young women. We excluded women who were pregnant at the time of survey, and those who delivered their first baby after age 20. We also excluded those with incomplete information that was required for our study. Following the selection process, we obtained data set of 2,808 women. We subsequently checked the available data for outliers using an informal technique [18] (checking for abnormal BMI values based on a scatter diagram), and removed some subjects from analysis because their data may affect the interpretation of results [19, 20]. We eventually came up with a list of 2,743 young women who had delivered at least one child before age 20.

BDHS-2011 was conducted by way of two-stage stratified cluster sampling. In the first stage, the researchers selected 600 enumeration areas (EAs) with probability proportional to the EA size. Of these clusters, 207 EAs were from urban areas and 393 from rural areas. In the second stage, a systematic sampling method was used to select an average of 30 households for each EA. This would provide statistically reliable estimates of key demographic and health variables for the whole country, including all the seven divisions, covering both the urban and rural areas. The BDHS is a part of the worldwide Demographic and Health Surveys program. The BDHS-2011 was conducted under the authority of the National Institute of Population Research and Training (NIPORT) of the Ministry of Health and Family Welfare. The project was conducted by Mitra and Associates, a Bangladeshi research firm based in Dhaka. ICF International from Maryland, United States (US), provided technical support as part of its international Demographic and Health Surveys program (MEASURE DHS). United States Agency for International Development (USAID) provided the financial support for the project. The sampling technique, survey design, survey instruments, measuring system, quality control, ethical approval and subject consent for the 2011 BDHS have been described elsewhere [21]. BDHS-2011 collected socio-demographic, health and lifestyle information from each of their selected subject from July 8, 2011 to December 27, 2011. In addition, body height and weight were measured for all subjects.

### Outcome variable

The outcome variable of this study was nutritional status, and it was measured by BMI. BMI was defined and calculated as the ratio of weight in kilograms to height in meters squared. The BMI was classified according to most widely used categories for the adults: (i) underweight (under-nutrition) (BMI ≤ 18.5 kg/m^2^), (ii) normal weight (18.5 < BMI <25 kg/m^2^), (iii) overweight (25 ≤ BMI < 30 kg/m^2^) and (iv) obese (BMI ≥ 30 kg/m^2^) [16, 22, 23]. The subjects were also classified on the basis of chronic energy deficiency (CED) grades as follows: (i) grade III (severe thinness) (BMI < 16.0 kg/m^2^), (ii) grade II (moderate thinness) (16.0 ≤ BMI <17.0 kg/m^2^), (iii) grade I (mild thinness) (17.0 ≤ BMI < 18.5 kg/m^2^) [17, 24].

### Independent variables

Various socio-economic and demographic factors were used in this study as independent variables, and they included : type of place of residence, respondent’s (woman) educational level, partner’s (husband) educational level, respondent’s occupation, partner’s occupation, wealth index, type of delivery, place of delivery, total number of children ever born, age at first marriage, and respondent age at first birth. More detail on the definition of these variables is available in the BDHS-2011 survey report [21].

### Statistical analysis

Descriptive statistics was done for calculating prevalence of underweight, normal weight, overweight and obese among early childbearing mothers. Chi-square test was utilized in this study for selecting significant independent factors for logistics regression models. Finally, binary multiple logistic regression was used to examine the relative importance of socio-demographic factors on early childbearing mothers’ health. In this model, category of body size (BMI) was considered as a dependent variable coded as 0 = normal weight and 1 = underweight. The underlying multiple logistic regression models corresponding to each variable is:

$$ \log \left(\frac{p}{1-p}\right)={\upbeta}_0+{\upbeta}_1{\mathrm{X}}_1+{\upbeta}_2{\mathrm{X}}_2+{\upbeta}_3{\mathrm{X}}_3+{\upbeta}_4{\mathrm{X}}_4+{\upbeta}_5{\mathrm{X}}_5+{\upbeta}_6{\mathrm{X}}_6+{\upbeta}_7{\mathrm{X}}_7+{\upbeta}_8{\mathrm{X}}_8+{\upbeta}_9{\mathrm{X}}_9+{\upbeta}_{10}{\mathrm{X}}_{10}+{\upbeta}_{11}{\mathrm{X}}_{11} $$

where, *p =* the probability of underweight (coded 1)

1-*p =* the probability of normal (coded 0)

X_1_ = place of residence (coded; urban = 0, rural = 1)

*X*_2_ = respondent’s (woman) educational level (coded; no education = 0, school education = 1, higher education = 2)

X_3_ = partner’s (husband) educational level (coded; no education = 0, school education = 1, higher education = 2)

X_4_ = respondent’s occupation (coded; housewife = 0, hard labor = 1)

X_5_ = partner’s occupation (coded; employed = 0, farmer/worker = 1)

X_6_ = wealth index (coded; poorest = 1, poorer = 2, middle = 3, richer = 4, richest = 5)

X_7_ = type of delivery (coded; non-caesarian = 0, caesarian = 1)

X_8_ = place of delivery (coded; hospital/clinic = 0, home = 1)

X_9_ = total number of children ever born

X_10_ = age at first marriage

X_11_ = respondent age at first birth, and

β_0_ = intercept term, and β_1_, β_2_,…,β_11_ are unknown coefficients.

Multicollinearity problem among the predictor variables were checked by standard error (SE) [25]. If the magnitude of the SE is approximately 0.001–5.0 [25], suggested that there is no evidence of Multicollinearity problem. All the statistical analyses were carried out using Statistical Package for Social Scientists (IBM SPSS version 22.0) software.

## Results

After we analysed the data set of 2,743 young non-pregnant young Bangladeshi mothers (age 24 or younger) who delivered their first child before age 20, we noted that the mean age of the mothers was 20.49 ± 2.37 years (95 % CI: 20.40–20.58). The mean number of children per mother was 1.60 ± 0.76. More than half (53.8 %) of them had one child, about one third (34.6 %) had two children, and the rest (11.6 %) had two and more children. Mean weight of all mothers was 45.97 ± 8.01 kg (95 % CI: 45.67–46.27), ranging from 28.00 to 104.20 kg. Mean height of the mothers was 150.87 ± 5.48 cm (95 % CI: 150.66–151.07), ranging from 112.10 to 197.20 cm. BMI varied from 13.09 kg/m^2^ to 38.13 kg/m^2^, with a mean of 20.16 ± 3.07 kg/m^2^ (95 % CI: 20.04–20.27) (Table 1). The age at first childbirth in these women varies from 12 to 19 years, with the mean being 16.54 ± 1.67 years (95 % CI: 16.48–16.60).

**Table 1:** Descriptive statistics for age, age of first birth, weight, height and BMI of early childbearing young mothers in Bangladesh (*n =* 2743)

Variable	Mean	SD	SE	95 % CI for mean		Mini-mum	Maxi-mum
				Lower	Upper		
Age (year)	20.49	2.377	0.045	20.40	20.58	13	24
Age of first birth (year)	16.54	1.679	0.032	16.48	16.60	12	19
Weight(kg)	45.97	8.014	0.153	45.67	46.27	28.00	104.20
Height (cm)	150.87	5.482	0.104	150.66	151.07	112.10	197.20
BMI (kg/m^2^)	20.16	3.075	0.058	20.04	20.27	13.09	38.13

**Fig. 1:**
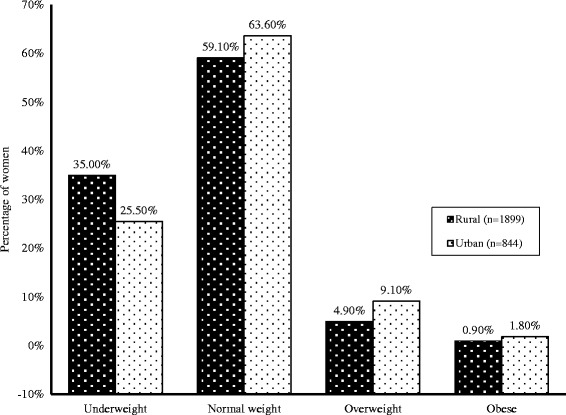
Difference between urban and rural in the percentage of underweight and obese mothers in Bangladesh

Based on BMI categories, more than half (60.5 %) of the mothers had normal weight, and about one third (32.1 %) of them were underweight. There were relatively few mothers who were overweight (6.2 %) or obese (1.2 %) (Table 2), and they were mostly from the urban areas (Fig. 1). When we considered mothers who had CED (same criteria as underweight based on BMI categories: BMI ≤ 18.5 kg/m^2^), 11.9 % were grade III (severe), 25.9 % were grade II (moderate), and 62.2 % were mild (grade I) (Table 2).

**Table 2:** Frequency distribution of BMI category and chronic energy deficiency of early childbearing mothers (*n =* 2743)

Variables	n (%)
BMI category	
Underweight (BMI ≤ 18.5 kg/m^2^)	879 (32.1 %)
Normal weight (18.5 < BMI < 25 kg/m^2^)	1660 (60.5 %)
Overweight (25 ≤ BMI < 30 kg/m^2^)	171 (6.2 %)
Obese (BMI ≥30 kg/m^2^)	33 (1.2 %)
CED category	
CED grade III (severe thinness) (BMI < 16.0 kg/m^2^)	104 (11.9 %)
CED grade II (moderate thinness) (16.0 kg/m^2^ ≤ BMI < 17.0 kg/m^2^)	228 (25.9 %)
CED grade I (mild thinness) (17.0 kg/m^2^ ≤ BMI ≤ 18.5 kg/m^2^)	547 (62.2 %)

Women from the rural areas were more likely to be underweight (38.8 %) compared to those from urban areas (25.5 %). Those with lower level of education (39.9 %) were more likely to be underweight compared to those who had higher level of education (30.3 %). Level of education for the husband also showed similar pattern of influence on the nutritional status of these women. Family income was another factor that influenced the BMI. Our study showed that women from poor households were more likely (39 %) to be underweight compared with those not from poor households (26.1 %). We also noted that women who were not working (housewives) were less likely to be underweight compared to those who had to work (32.4 % vs 49.0 %). On the contrary, women with husbands who were unemployed were more likely to be underweight compared to those with working husbands (37.7 vs 30.8 %). Chi-square test showed that the association of all these factors with low BMI (being underweight) were statistically significant (*p <* 0.001).

When we look at the method and place of delivery their child, we noted that mothers who delivered at home had higher risk of being underweight compared to those who delivered in hospitals or clinics (36.4 % vs 28.7 %). The association of place of delivery and underweight was significant (*p <* 0.001). Those who delivered naturally (non-Caesarean section) were more likely to be underweight (35.3 % vs 27.7 %) since most of them were home deliveries. The association of type of delivery and underweight was statistical significant (*p <* 0.05). Women who were married before age 17 were more likely to be underweight compared to those who were married later (35.2 % vs 31.1 %). In addition, more than one third of women who delivered their first child before age 17 were underweight, and this was much higher than those who delivered their first child at an older age (36.8 % vs 32.1 %). We also noted that women who had more than two children were more likely to be underweight than those who had only one or two children (41.4 % vs 33.5 %). The association between all these factors and underweight was statistically significant (*p <* 0.05) (Table 3).

**Table 3:** Association between underweight and socio-economic, and demographic factors of early childbearing Bangladeshi mother (BDHS-2011) (*n =* 2539)

Variable	Group	Underweight n (%)	*χ* ^2^-value	*p-*value
Place of residence	Urban	215 (25.5)	44.09	<0.001
	Rural	659 (38.8)		
Mother education level	Up to primary	431 (39.9)	25.04	<0.001
	Secondary and higher	443 (30.4)		
Partner education level	Up to primary	560 (38.5)	25.52	<0.001
	Secondary and higher	314 (28.9)		
Respondent’s occupation	Housewives	722 (32.4)	33.39	<0.001
	Involved with hard labor	152 (49.0)		
Partner’s occupation	Employee	373 (30.8)	13.25	<0.001
	Non-employee	501 (37.7)		
Wealth index	Poor	638 (39.0)	43.39	<0.001
	Rich	236 (26.1)		
Place of delivery	Respondent home	688 (36.4)	12.38	<0.001
	Hospital/Clinic	186 (28.7)		
Type of delivery	Non-caesarean	793 (35.3)	6.33	0.011
	Caesarean	81 (27.7)		
Mother age at first marriage	Lowest through 16 years	730 (35.2)	4.27	0.039
	17 years and above	144 (31.1)		
Mother age at first birth	Lowest through 16 years	460 (36.8)	6.31	0.012
	17 years and above	414 (32.1)		
Total children ever born	1–2	751 (33.5)	7.28	0.007
	3 and above	123 (41.4)		

Since there were very few overweight and obese women in our sample population (6.2 % and 1.2 % respectively), we decided to exclude them from *χ*^2^-test and logistic regression model. We considered underweight as 1 (reference case) and normal as 0 (non-reference case), and used them as dependent variable in this binary model. Only variables that demonstrated significant association were considered as independent variables in this model. The logistic regression coefficient and odds ratio showed that women who came from rural areas had a 1.478 times [95 % CI: 1.23–1.78; *p <* 0.01] higher chance to be underweight, as compared to those who came from urban areas. Uneducated mothers were 4.169 [95 % CI: 2.10–8.26; *p <* 0.01] times more likely to be underweight compared to educated mothers, and those with only secondary education were 2.997 times [95 % CI: 1.57–5.72; *p <* 0.01] more likely to be underweight compared to mothers with higher levels of education. Similar observation was noted when we analysed the risk of these women being underweight with education levels of their partner or husband (Table 4). Working mothers would have 2.085 times [95 % CI: 1.58–2.55; *p <* 0.01] higher risk of being underweight compared to mothers who were housewives. On the other hand, women whose partner was unemployed would have 1.204 times [95 % CI: 1.02–1.47; *p <* 0.05] higher risk of being underweight compared to women whose partner were working as farmer or worker. When we look at the economic background, risk of being underweight for women from poorest, poor, middle and rich families would be 3.303 times [95 % CI: 2.44–4.47; *p <* 0.01], 2.104 times [95 % CI: 1.55–2.86; *p <* 0.01], 1.944 times [95 % CI: 1.43–2.65; *p <* 0.01], and 1.870 times [95 % CI: 1.36–2.56; *p <* 0.01] higher than those from richest family respectively. The risk of being underweight for mothers who delivered naturally was 1.258 times [95 % CI: 1.03–1.85; *p <* 0.05] higher than those who delivered by Caesarian sections. Mothers who delivered at home had 1.290 times [95 % CI: 1.06–1.57; *p <* 0.05] higher risk of being underweight compared to mothers who delivered at hospitals or clinics. Young mothers with more than 2 children would have 1.41 times [95 % CI: 1.11–1.79; *p <* 0.01] higher risk to be underweight. On the other hand, age at first marriage [OR = 0.953, 95 % CI: 0.91–0.96; *p <* 0.05] and age at first childbirth [OR = 0.960, 95 % CI: 0.92–0.98; *p <* 0.05] were negatively related to being underweight, and the risk would reduce with the increasing age (Table 4).

**Table 4:** Effect of selected socio-economic and demographic characteristics on young mothers’ nutritional status (*n =* 2539)

Variable	^*^ *p-*value	OR ^a^ (95 % CI for odds ratio)
Place of Residence		
Rural (Ref. Urban)	*p <* 0.01	1.478 (1.23, 1.78)
Respondent education level		
No educated (Ref. Higher)	*p <* 0.01	4.169 (2.10, 8.26)
Secondary (Ref. Higher)	*p <* 0.01	2.997 (1.57, 5.72)
Partner education level		
No educated (Ref. Higher)	*p <* 0.01	2.417 (1.68, 3.48)
Secondary (Ref. Higher)	*p <* 0.01	1.851 (1.32, 2.61)
Respondent’s Occupation		
Hard labor mother (Ref. Housewife)	*p <* 0.001	2.085 (1.58, 2.55)
Partner’s Occupation		
Farmer/Worker (Ref. Employed)	*p <* 0.05	1.204 (1.02, 1.47)
Businessman (Ref. Employed)	0.302	0.878 (0.86, 1.13)
Wealth Index		
Poorest (Ref. Richest)	*p <* 0.01	3.303 (2.44, 4.47)
Poor (Ref. Richest)	*p <* 0.01	2.104 (1.55, 2.86)
Middle (Ref. Richest)	*p <* 0.01	1.944 (1.43, 2.65)
Rich (Ref. Richest)	*p <* 0.01	1.870 (1.36, 2.56)
Type of delivery		
Non-caesareans (Ref. Cesarean)	*p <* 0.05	1.258 (1.03, 1.85)
Place of Delivery		
Home (Ref. Hospital/Clinic)	*p <* 0.05	1.290 (1.06, 1.57)
Total Children Ever Born		
3 & more children (Ref. 1–2 children)	*p <* 0.01	1.141 (1.11, 1.79)
Age at First Marriage	*p <* 0.05	0.953 (0.91, 0.96)
Age at First Birth	*p <* 0.023	0.960 (0.92, 0.98)

Underweight was considered as reference category for dependent variable (underweight =1, normal weight =0)

Hosmer-Lemeshow test, (*p =* 0.96), Pearson Chi-square & Sig., (*p <* 0.001) and

classification table (overall correctly classified percentage = 87) were applied to check the model fitness

^a^Adjusted odds ratio

Note: *p-value (*p <* 0.01, 1 % and *p <* 0.05, 5 % level of significance)

## Discussion

Our study showed that the mean BMI of young childbearing women in Bangladesh was 20.16 kg/m^2^, and 32.1 % of these women were underweight. Very few young women in our study were noted to be overweight (6.2 %) or obese (1.2 %). Similar outcome pattern was observed in few other studies based on all Bangladeshi women [13, 15]. A large population study conducted in India reported a relatively similar finding where 31.2 % of their women were underweight, with only 9.4 % in overweight and 2.6 % in obese categories [17]. One local study that focused on women living in the slum areas in Bangladesh capital (Dhaka) reported that more than half (54 %) of these women were underweight, and concluded that the most likely contributing factor would be extreme poverty [26]. Among the underweight mothers (BMI ≤ 18.5), most of them had mild CED (grade I), more than one-fourth had moderate CED (grade II), and a relatively high percentage (11.9 %) suffered severe CED (grade III). Chronic malnutrition can retard the physiological developments of an individual, and this negative effect may be more prominent in young women in their reproductive age. It has been reported that although the difference in mortality rates between normal adult women and those with grade I CED was only about 1 % per year, but the percentage increased significantly in those with grade II and grade III CED (BMI <17.0 kg/m^2^) [27]. Our study showed that the risk of CED among rural women was higher than urban women, and this would most likely due to poverty and lack of resources on maternal health or general health education [21].

We noted that level of education was associated with nutritional status in young women of Bangladesh. Multivariate analyses indicated that the risk of being underweight for women who were either illiterate or with only primary education was higher than those with secondary or higher education. On the other hand, early childbearing was also linked with lower level of education, being undernourished and severe CED. Educated women were generally more conscious about the timing of childbearing, and many of them were well informed on adverse outcomes associated with early motherhood. Our study demonstrated that level of education of both these women and their husbands were important factors for nutritional status of our study subjects.

From the logistic regression analysis, we noted that the risk of being underweight of young women from rural area was higher than those from urban area. In Indian subcontinent, teenage marriage and early pregnancy are more common among rural compared to urban communities [28]. In addition to culture and religious factors, poverty has also been associated with teenage pregnancy [29]. Our analysis showed that wealth index influenced the BMI of Bangladeshi women, where poorer mothers had the higher chance to be undernourished. Poor countries such as Niger and Bangladesh had higher rates of teenage mothers compared with richer countries such as Switzerland and Japan [30]. Since more than 35 % of Bangladeshi women were poor, and 32 % were illiterate [21], we would expect these factors to contribute towards higher rates of early childbearing mothers in this country.

The present study demonstrated that age at first marriage, age at first birth, place of childbirth, and total number of children were important factors associated to nutritional status of young Bangladeshi women (Table 4). A local study by Hossain et al. also listed poor economic conditions, illiteracy, large family size, early age at marriage, early age at first delivery and lack of medical facilities as main factors contributing to the low BMI for married non-pregnant women staying in the rural areas [15]. By using the BDHS-2004 database, Khan and Kraemer [13] reported that age, education level, type of occupation, place of stay and marital status influenced the BMI of married non-pregnant women from the urban areas of Bangladesh. Based on the Nutritional Surveillance Project (NSP) conducted between 2000 and 2004, Shafique et al. reported that age, level of education, wealth index, place of residence were important factors that influence the BMI of women in this country [14].

Data used in this study was gathered by the BDHS-2011 that covered both the rural and urban populations, and our study indicated that early childbearing is still common and the practice is deeply entrenched in Bangladeshi culture. There were two other studies on the association of BMI with various socio- demographic factors in Bangladeshi women aged between 15 and 49 [13, 14]. In the current study, we focused on the subgroup aged 24 and below because they would represent the younger generation who will most likely respond to corrective measures by the authorities and public health organizations. According to the 65th World Health Assembly report [31], there were about 2 million girls under the age of 15 and 16 million girls aged between 15 to 19 years giving birth every year worldwide. About 95 % of all adolescent births occurred in low and middle income countries, and they were more likely staying in the poor rural areas and had little education. In developing countries, about 90 % of births in adolescents occur within marriage. The percentage was about 70–80 % in South America and in sub-Saharan Africa, but close to 100 % in Western Asia/Northern Africa, Central Asia, and South-Central and South-Eastern Asia [32]. The National Campaign to Prevent Teen Pregnancy of World Health Organization reported that career opportunity for many young women was reduced by earlier childbearing and failure to complete high school education [30]. Despite these obvious problems and undesirable consequences, most of the adolescent pregnancies in Bangladesh were pre-planned and highly valued [33]. With poverty and lack of education, the young families would not know the medical and health problems associated with their behaviour.

### Limitations of study

This study used secondary data derived from a national level cross-sectional survey where some of pertinent health indicator variables (like blood pressure, mortality as well as morbidity pattern) were not available. Despite the careful design and stratification of sample population, selection bias and reporting error might still be possible, and it was not known what women’s height and weight were taken before the pregnancy or immediate after the delivery. We only investigate the association between selected socio-economic and demographic factors with nutrition status that was represented by BMI. We were not able to cover physiological factors that may also be influenced by nutritional status of the women, like age at menarche [34], level of physical activities, level of energy intake [35], and behaviour patterns like dietary habits, smoking habits, weight goals, methods of weight-loss and body-shape perceptions [36]. Subsequent researchers may consider including these variables.

## Conclusion

About one third (32.1 %) of early childbearing mothers in Bangladesh were underweight, and 37.4 % of these underweight mothers were considered to have moderate (grade II) and severe (grade III) CED. Low level of education of both the women and their spouses, and poverty were factors associated with poor nutritional status, especially for those from the rural areas. Women who delivered naturally and those who delivered at home were more likely to ne underweight, and this was also applicable for those with more than two children at the time of study. Our study also noted that early marriage and early childbearing were still commonly practiced in Bangladesh. Our study indicated that poor nutrition remained a serious problem among young childbearing mothers in Bangladesh, and we hope that information derived from this study would be able to help relevant authorities to plan remedial actions.

## Abbreviations

BDHS: Bangladesh Demographic and Health Survey
BMI: Body mass index
CED: Chronic Energy Deficiency
EAs: Enumeration areas
HDI: Human Development Indicators
NIPORT: National Institute of Population Research and Training
NSP: Nutritional Surveillance Project
USAID: United States Agency for International Development
